# Increasing the doping efficiency by surface energy control for ultra-transparent graphene conductors

**DOI:** 10.1038/s41598-017-09465-x

**Published:** 2017-08-22

**Authors:** Kai-Wen Chang, Ya-Ping Hsieh, Chu-Chi Ting, Yen-Hsun Su, Mario Hofmann

**Affiliations:** 1 Department of Material Science and Engineering, National Cheng Kung University, Tainan, 70101 Taiwan; 2 Institute of Atomic and Molecular Sciences, Academia Sinica, Taipei, 10617 Taiwan; 3 Department of Physics, National Taiwan University, Taipei, 106 Taiwan

## Abstract

Graphene’s attractiveness in many applications is limited by its high resistance. Extrinsic doping has shown promise to overcome this challenge but graphene’s performance remains below industry requirements. This issue is caused by a limited charge transfer efficiency (CTE) between dopant and graphene. Using AuCl_3_ as a model system, we measure CTE as low as 5% of the expected values due to the geometrical capacitance of small adsorbate clusters. We here demonstrate a strategy for enhancing the CTE by a two-step optimization of graphene’s surface energy prior to AuCl_3_ doping. First, exposure to UV ozone modified the hydrophilicity of graphene and was found to decrease the cluster’s geometric capacitance, which had a direct effect on the CTE. Occurrence of lattice defects at high UV exposure, however, deteriorated graphene’s transport characteristics and limited the effectiveness of this pretreatment step. Thus, prior to UV exposure, a functionalized polymer layer was introduced that could further enhance graphene’s surface energy while protecting it from damage. Combination of these treatment steps were found to increase the AuCl_3_ charge transfer efficiency to 70% and lower the sheet resistance to 106 Ω/γ at 97% transmittance which represents the highest reported performance for doped single layer graphene and is on par with commercially available transparent conductors.

## Introduction

Graphene, a two-dimensional carbon allotrope, is anticipated to be an enabling material for flexible and transparent electronics^1^. Despite significant advances in the synthesis of high quality material, graphene’s performance is still below the industry standards for transparent conducting films (TCFs)^2–4^. This shortcoming is caused by graphene’s low intrinsic carrier concentration due to its unique band structure^5^. A common method to enhance graphene’s conductivity is through extrinsic doping where oxidizing or reducing agents are introduced to remove or add charges to the graphene. This method combines easy scalability and high performance compared to alternative approaches such as graphene/mesh hybrids^6^ and intercalation compounds^7^. A wide variety of materials have been employed for extrinsic doping, such as nitric acid^8^, silver nitrate^9^, iron chloride^7^, gold chloride^10^ and ammonia gas^11^. While these materials enhance the carrier concentration of graphene, they also decrease graphene’s transparency. To capture the simultaneous variation in transmittance and resistance, a figure of merit (FOM) is commonly employed^12^ that represents the ratio of graphene’s conductivity at DC and optical frequencies and should be maximized.

1 $$FOM=\frac{{\sigma }_{DC}}{{\sigma }_{Op}}=\frac{377{\rm{\Omega }}\,}{2{R}_{s}(\frac{1}{\sqrt{T}}-1)},$$

where Rs is the sheet resistance and T is the sample’s transparency. The FOM can be related to atomic parameters^12^ and depends on the dopant’s molecular light extinction coefficient *ε* and graphene’s carrier mobility *μ*_*G*_ according to

2 $$\frac{{\sigma }_{DC}}{{\sigma }_{Op}} \sim \frac{{\mu }_{g}}{{\varepsilon }_{D}}\frac{n}{c},$$

where n is the number of charges in the graphene and c is the concentration of dopant. Thus, for a given material combination, graphene’s performance is controlled by the amount of charges that transfer from the dopant to the graphene, a parameter we will term charge transfer efficiency (CTE). When focusing on the well-established wet-chemical doping process using gold (III) chloride (AuCl_3_)^2, 13–17^, we found no report of an FOM larger than 50 which does not satisfy requirements for many current-driven applications^12, 18^. The question arises if there is a fundamental limit for the FOM as previously suggested^12^ or if optimization of the doping process could increase the FOM enough to compete with traditional TCF materials.

Here we demonstrate that control over graphene’s surface energy can enhance the efficiency of charge transfer between dopants and graphene. A strong dependence of the AuCl_3_ charge transfer efficiency on surface energy was found to be due to geometry-induced work function changes of the dopant. To maximize the CTE, we devised a multistep pretreatment that enhances graphene’s surface energy. Exposure to UV-generated ozone showed a clear trend between graphene’s hydrophilicity and the amount of transferred charge which was ascribed to the formation of functional groups in the graphene basal plane. The extent of CTE-increase is limited by the onset of destructive oxidation of graphene at prolonged UV exposure. To minimize the impact of defects on carrier transport, a polymer layer was introduced on top of the graphene and functional groups were produced in this film. Enhanced thermal scission was found to further enhance graphene’s surface energy and thus treated graphene showed an improved CTE of 70% and exhibited the highest reported figure of merit for doped graphene in excess of 110 while retaining a transparency of 97%. These features make the presented approach promising for industrial applications of graphene-based transparent conducting films.

## Experimental

Single layer graphene was grown by chemical vapor deposition on copper foil(Alfa Aesar 31882) following previous reports^19^. Briefly, copper foil is annealed at 1000 °C under 10 sccm hydrogen gas flow for 30 minutes, then graphene is grown under a flow of 40 sccm methane and 100 sccm hydrogen gas flow for one hour before being cooled down to room temperature under a flow of 10 sccm H_2_. After growth, graphene is transferred onto quartz or SiO_2_/Si substrates using established procedures^20^.

AuCl_3_ solution was produced by dissolving AuCl_3_ (334049 Sigma-Aldrich) in Nitromethane solution following previous reports^17^. This solution was cast onto a graphene device at room temperature using a micropipettor and left overnight for drying. Poly(methyl methacrylate) (Microchem A4) was spin coated at 2500 rpm for 1 minute. Spin curves predict a film thickness of approximately 250 nm under these conditions. UV illumination was conducted in a homebuilt chamber using a light source with a power of 13.3 mW.

Raman spectroscopy (MRI, ProtrusTech) was carried out using a 532 nm laser source. The sample’s sheet resistance and Hall carrier concentration were measured in van-der-Pauw geometry using an Agilent B2900A source meter and a homebuilt probe station. Work function was measured through Kelvin probe measurements. Haze measurements were carried out in an integrating sphere using an Evolution 220 Spectrophotometer.

## Results and Discussions

We carry out doping by casting 100 µl of AuCl_3_ solution on graphene. This process^16^ is expected to produce a reactive species of AuCl_4_- ions that remove electrons from the graphene resulting in p-type doped graphene and neutral gold clusters according to

3 $$AuC{l}_{4}^{-}+3e\to A{u}^{0}+4C{l}^{-}$$

**Figure 1:**
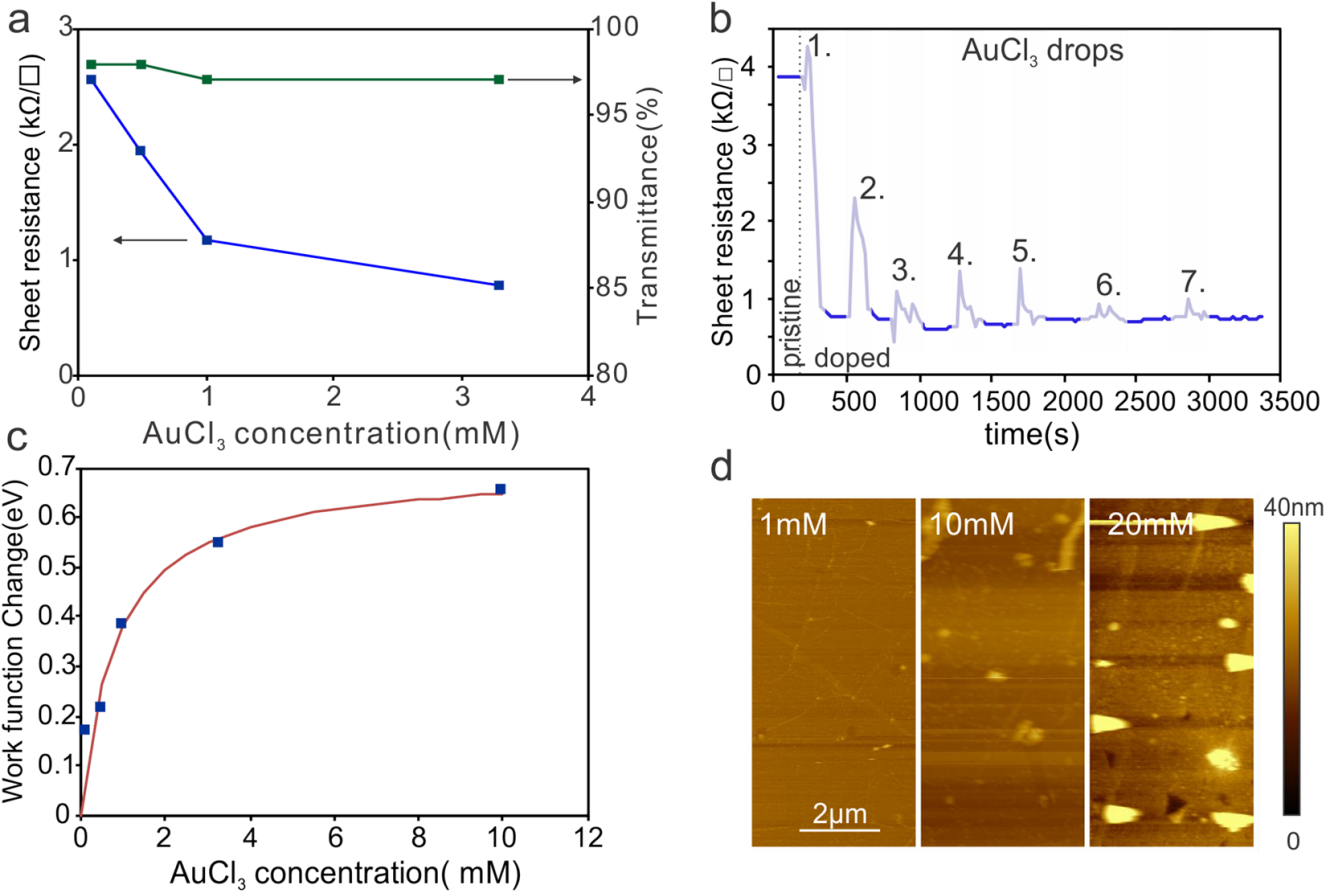
(**a**) Sheet resistance and transmittance vs. AuCl_3_ concentration, (**b**) sheet resistance after repeated dropping of AuCl_3_ solution onto the sample, (**c**) work function change vs. AuCl_3_ concentration with fit to model as explained in the text, (**d**) representative AFM images of graphene with different concentrations of AuCl_3_ solution.

It is observed that a higher AuCl_3_ concentration shows a decrease in sheet resistance in agreement with previous reports^16^ (Fig. 1(a)) which had been previously attributed to an increase in the amount of AuCl_3_ deposited onto the graphene. This explanation is challenged, however, by the observation that the transmittance does not decrease significantly with increasing AuCl_3_ concentration (Fig. 1(a)). To identify if there is a relation between the amount of AuCl_3_ deposited and the achievable sheet resistance, we repeated the casting process several times while monitoring the resistance *in-situ* (Fig. 1(b)). We observe a 5fold decrease in resistance after the first droplet of AuCl_3_ solution is deposited but no significant change in resistance after the second droplet is deposited. Casting of a 6x higher amount of AuCl_3_ was found to have no appreciable effect on the achievable resistance which suggests a self-limiting doping process that is independent of the deposited volume.

Surprisingly, we find a clear dependence of the achievable sheet resistance on the concentration of AuCl_3_ in solution (Fig. 1(c)). To understand this trend, we investigate graphene’s work function change, as extracted from its Hall carrier concentration^21^. It can be seen from Fig. 1(c) that graphene’s work function increases with AuCl_3_ concentration indicating that this parameter is controlling the doping of graphene. To explain the observed concentration-dependent doping effect, we model the charge transfer between AuCl_3_ and graphene. This system can be represented by two capacitors that supply/accept charges in contact with each other until their initial voltage is equilibrated. The trend of Fig. 1(c) is very well captured by a concentration dependent capacitance of AuCl_3_ (A detailed derivation of the model is provided in the Supplementary Discussion). This observation indicates that at high AuCl_3_ concentrations charge transfer between graphene and AuCl_3_ proceeds until graphene has reached the same work function as the dopant. However, for lower AuCl_3_ concentrations, a lower amount of available charges from AuCl_3_ will only raise graphene’s work function to an intermediate level.

For high concentrations the work function change is found to saturate at approximately 0.7 eV. To identify the physical meaning of this value, we investigate the difference in reduction potential between graphene (0.22 V) and AuCl_4_- ions (1 V) that form during the doping process^16^. Based on this simple picture the highest achievable work function shift upon reduction is 0.78 eV which agrees well with the experimental value. Considering this theoretical reduction potential difference we can estimate a doping concentration of ${n}_{compl.}=5\times {10}^{13}c{m}^{-2}$ for complete charge transfer between AuCl_3_ and graphene. We can thus quantify the charge transfer efficiency (CTE) as the fraction of actual chare transfer compared to the complete transfer case.

4 $$CTE=n(c)/{n}_{compl.}$$

The thus extracted CTE is only 5% for 0.1 mM but 50% for 3.3 mM AuCl_3_ at similar transparencies which highlights the importance of this parameter for enhancing the performance of graphene. In order to identify the origin of the low CTE at low carrier concentrations, we carried out AFM imaging of samples after AuCl_3_ treatment. We find that higher AuCl_3_ concentrations form larger clusters on graphene (Fig. 1(d)), in agreement with previous reports^22^. Consequently, there seems to be a relation between the cluster size and the CTE. Indeed, previous reports found that gold nanoparticles exhibit a radius-dependence on apparent work function due to geometry-induced changes in the capacitance of Au-clusters^23^. We conclude that an increasing AuCl_3_ concentration is thus improving the CTE by producing larger particles with larger capacitance that are easier to discharge. Too high AuCl_3_ concentrations, however, will not only increase the CTE but also the light absorption and thus reduce the FOM (Supplementary Figure S1). Therefore, other means of increasing the dopant cluster size have to be pursued to further enhance the CTE.

**Figure 2:**
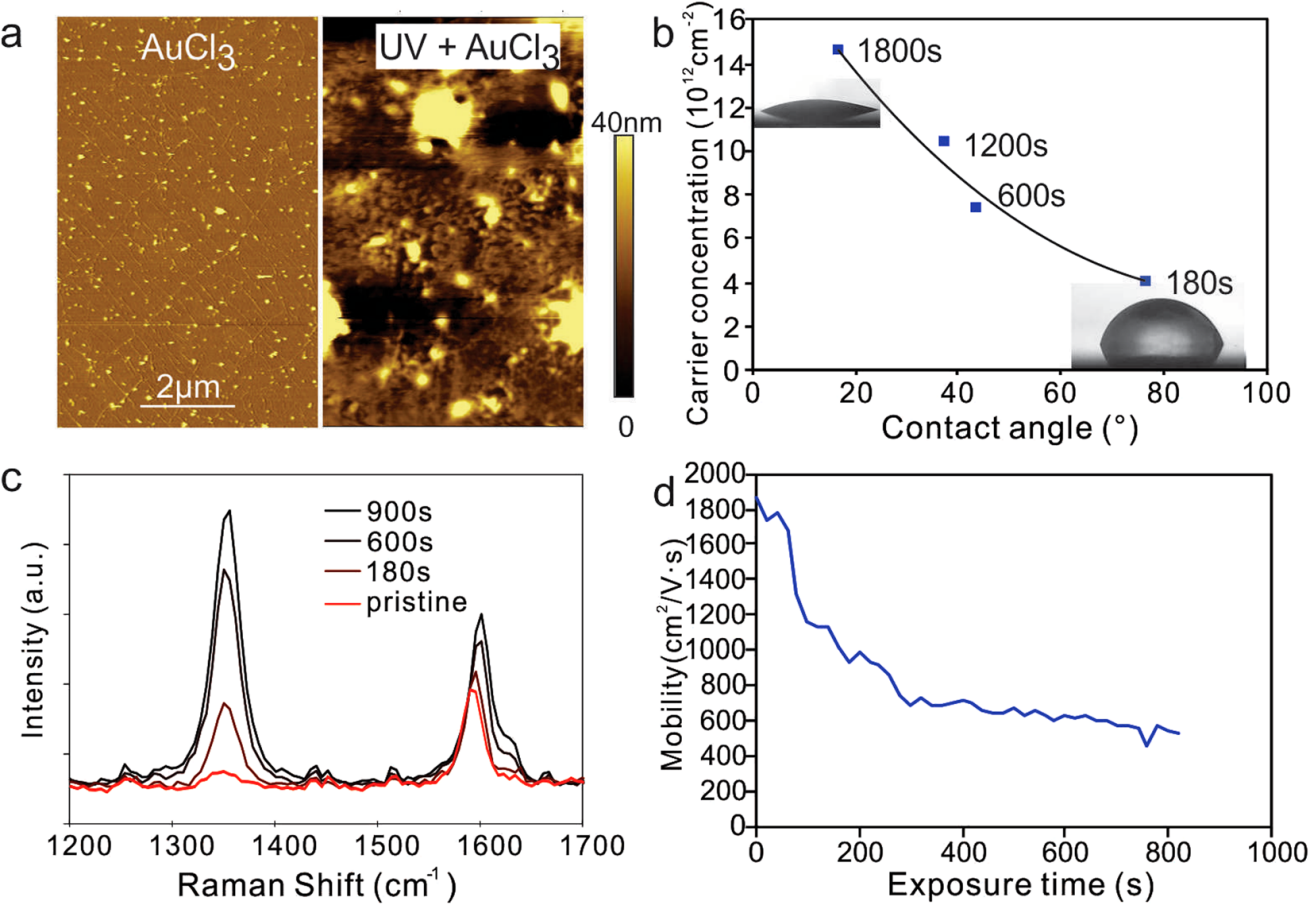
(**a**) Representative AFM images of graphene after AuCl_3_ deposition with(right) and without(left) UV exposure, (**b**) Carrier concentration vs. contact angle for different UV exposure durations (**c**) Raman spectra at different UV exposure, (**d**) carrier mobility vs. UV exposure time.

The size of a cluster is determined by the competition between the surface energy of the support and the surface tension of the cluster^24, 25^. Thus tuning the surface energy of the graphene could serve as another method to increase the cluster size^26^. One common method to vary the surface energy is through UV-generated ozone^27^. We therefore exposed our samples to UV light and then immediately deposited AuCl_3_ solution. The resulting sample morphology is markedly different from samples without UV exposure (Fig. 2(a)). AFM reveals cluster dimensions that are twice as large as the untreated sample which corroborates the relation between surface energy and cluster size.

Contact angle measurements were conducted to quantify the relation between surface energy and CTE. We observe that a high surface energy (as indicated by a low contact angle) will exhibit a higher amount of transferred charges (Fig. 2(b)). This result confirms that graphene’s surface energy is indeed controlling the CTE and the surface energy should be maximized for optimal doping.

We try to identify the underlying mechanism of the surface energy enhancement upon UV exposure. Raman spectra show an increasing D-band intensity that suggests an increasing defectiveness of graphene with UV exposure. (Fig. 2(c)). Moreover, the extracted D’/D ratio (6.7) is indicative of vacancy-like defects^28^. Previous reports showed that the UV exposure of graphene will produce functional groups, such as hydroxyl groups^29^, that enhance its surface energy^30^ but such functional groups simultaneously increase the carrier scattering^31^. We indeed observe that graphene’s carrier mobility decreases significantly upon prolonged UV exposure (Fig. 2(d)). Consequently, the enhancement in the CTE by UV exposure is offset by the decrease in graphene’s conductivity and another method for introducing functional groups has to be found.

**Figure 3:**
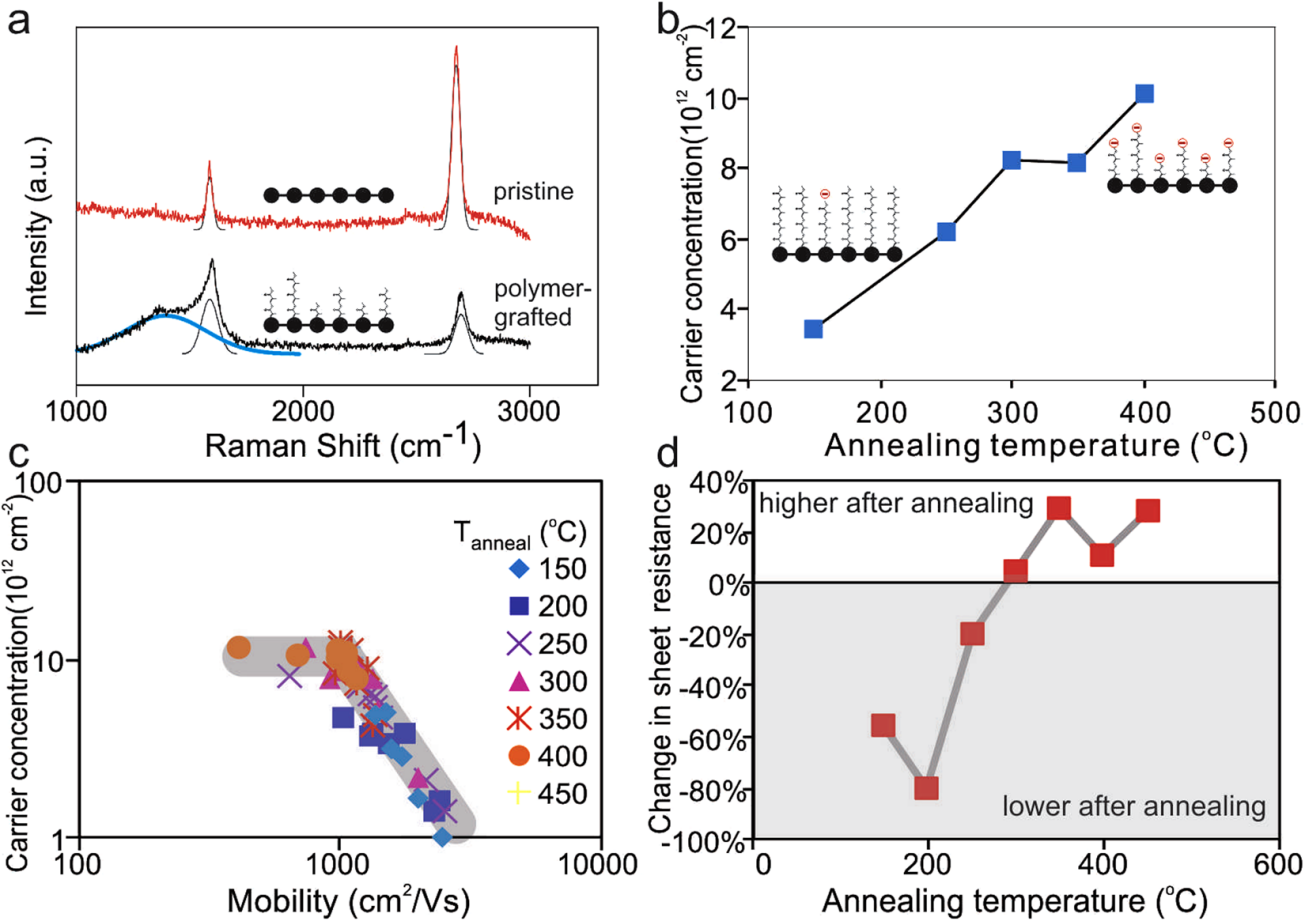
(**a**) Representative Raman spectra of PMMA coated graphene with and without thermal treatment, (**b**) carrier concentration vs. annealing temperature (inset) schematic of thermal scission and functionalization of sacrificial layer on graphene, (**c**) carrier concentration vs. carrier mobility for samples annealed at different temperatures, (**d**) difference in sheet resistance between annealed and pristine samples for different annealing temperatures.

The increasing hydrophilicity in graphene had been related to the splitting of water on graphene under UV exposure and this process was found to preferentially occur on the sites of lattice defects in graphene^30^. Thus, enhancements in hydrophilicity can be achieved by providing functional groups that emulate such lattice defects. We produce such functional groups by the thermal scission of Poly(methyl methacrylate) (PMMA). The polymer is known to preserve graphene’s high carrier mobility^32^ and can be decomposed into short oligomers by thermal annealing^33^. For this purpose, annealing of the substrate/graphene/PMMA stack at high temperatures in a forming gas of 600sccm H_2_ and 400sccm Ar for 150 minutes was carried out. Raman spectroscopy shows the occurrence of a broad peak around the D-band that had been previously identified as a fingerprint of fragmented PMMA after thermal treatment (Fig. 3(a)) ^34^. AFM images show little residue after annealing (Suppl. Figure S3) in agreement with previous reports that the oligomers are in the form of a continuous thin film^34^.

To investigate the effect of temperature on bond-scission, graphene’s carrier concentration was analyzed. Upon scission, a charged radical occurs at the end of the remaining chain^34^. Thus, the charge induced in the graphene represents a measure of the radical concentration. We find that the amount of induced charge increases with annealing temperature which supports the model of temperature induced functionalization (Fig. 3(b)). The enhanced thermal scission at high annealing temperatures, however, was found to not only increase graphene’s charge density but also result in more scattering. This observation was quantified in Fig. 3(c) where graphene’s carrier mobility was plotted against the carrier concentration. At low annealing temperatures an inverse relation was extracted that is indicative of short-range carrier scattering at varying doping^31^. If the annealing temperature exceeded 300 °C, however, the carrier concentration stagnated while the carrier mobility continued decreasing, resulting in a horizontal line. This behavior suggests the formation of lattice defects in graphene which deteriorates carrier transport. This conclusion is supported by measurements of graphene’s resistance change after annealing. Compared to pristine graphene, annealing can reduce graphene’s resistance if temperatures below 300 °C are chosen (Fig. 3(d)). Annealing PMMA under these conditions was shown to reduce the contact angle from 75° for pristine graphene to 35° for oligomer-covered PMMA which is consisted with the reduction in contact angle for bare SiO_2_ from 70° to 10° and demonstrates the hydrophilic character of the oligomers.

**Figure 4:**
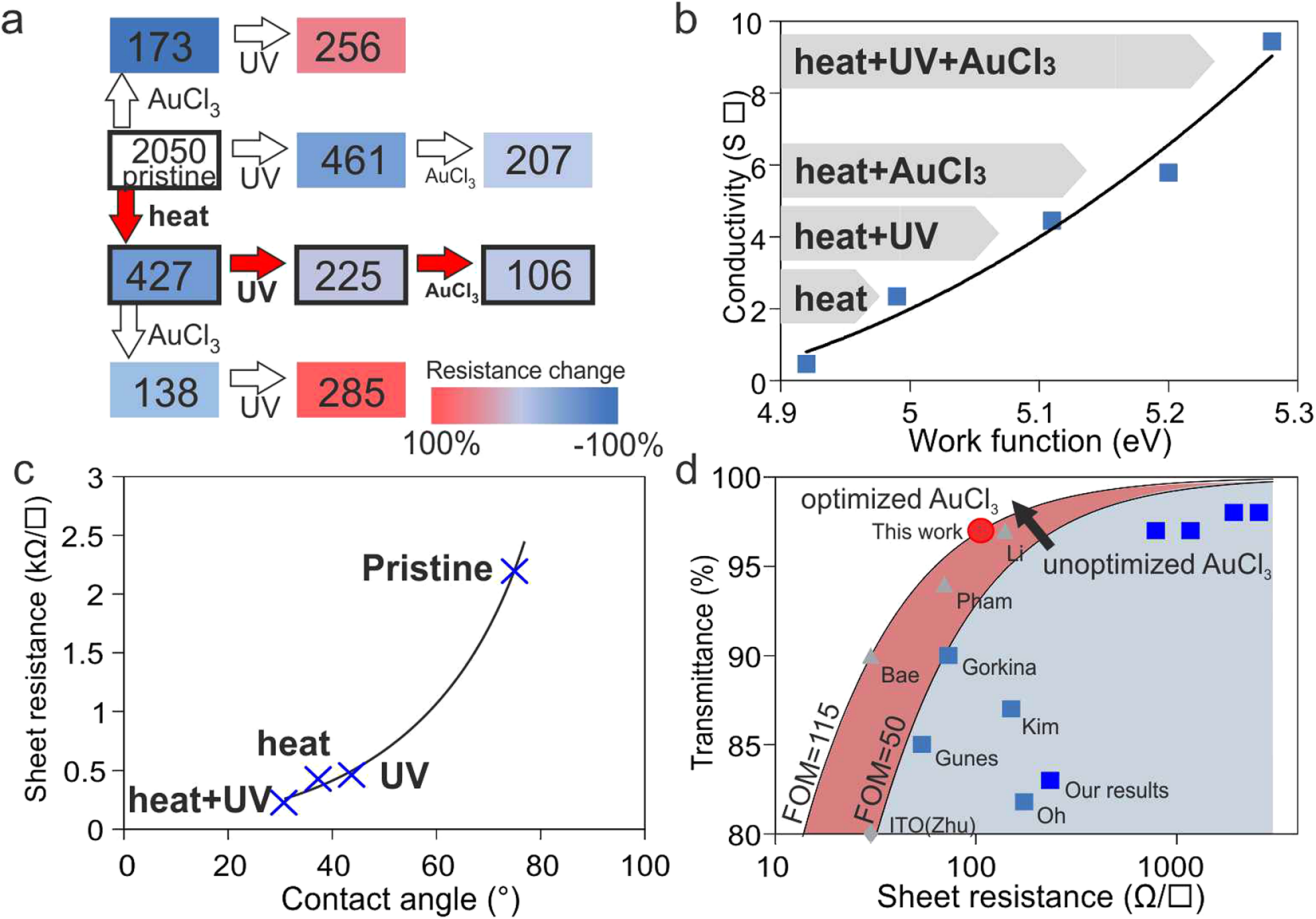
(**a**) Schematic of different doping sequences and resulting resistance after each step, (**b**) work function and conductance after different pretreatment steps, (**c**) contact angle vs. resistance for different doping steps (**d**) comparison of graphene performance before and after optimization with previous reports for AuCl_3_ doping (squares)^2, 15–17^, other doping methods (triangles)^35–38^, and ITO (diamond)^37^.

We now combine all the introduced pretreatment steps (Suppl. Figure S4). First, a polymer layer is deposited and broken down by heat-induced conversion. Then, functional groups are introduced on the polymer by UV exposure. *In-situ* measurements show a qualitatively similar evolution of the graphene mobility under UV exposure with and without the presence of the polymer (Suppl. Figure S5), which suggests that the previously described functionalization process still occurs. Finally, AuCl_3_ is deposited on the thus prepared structure. We observe that this sequence is the only one that lowers the sheet resistance at every process step (Fig. 4(a)) which supports our hypothesis that the steps are building upon one another.

Each step was found to significantly affect the work function of graphene as directly measured by a Kelvin probe. These results suggest that an enhanced CTE is the origin of the conductivity increase (Fig. 4(b)). Under optimized conditions we calculate a CTE of 70% according to equation 3. The associated conductivity enhancement was correlated with contact angle measurements after each pre-treatment step (Fig. 4(c)). We observe an exponential dependence of graphene’s sheet resistance on the contact angle which indicates the sensitive dependence of graphene’s performance on an increased surface energy.

The potential of our approach is highlighted by the observed enhancement in the figure of merit. Graphene prepared by the describe sequence of treatment steps exhibits a sheet resistance of 106 Ω/γ at a transmittance of 97% (See Supplementary Figure S2. for the spectrum) which translates to a figure of merit of 116. This performance is significantly higher than AuCl_3_ doping without surface pretreatment as shown in Fig. 4(d) and is more than twice the FOM of any previous reported AuCl_3_ doping result. Furthermore, the achieved FOM represents the highest reported value for any doped single-layer graphene sample and is on par with triple-layer graphene champion devices. We furthermore carried out haze measurements and found that the average increase in haze for samples before and after AuCl_3_ doping is 1.1% and thus retains the advantageous properties of graphene-based transparent electrodes.

## Conclusions

In conclusion, we have demonstrated the enhancement of the graphene doping process by optimization of the charge transfer efficiency. This parameter was found to be limited by the geometric capacitance of small clusters and we designed a sequence of pretreatment steps that maximizes the dopant cluster size. Exposure to UV light was shown to increase graphene’s surface energy through introduction of functional groups and directly affect the CTE. To overcome the deterioration of the carrier transport by scatterers in the graphene lattice a sacrificial polymer layer was used as an anchor for functional groups. Combination of these steps increased the CTE to 70%, doubled AuCl_3_-doped graphene’s figure of merit, and produced doped single layer graphene with the highest reported performance. The presented approach of surface energy control for enhanced doping is applicable to many dopants and opens up a new route to increasing graphene’s potential for optoelectronic applications.

## Electronic supplementary material

Supplementary material
