# Supplementary material for: Increasing the doping efficiency by surface energy control for ultra-transparent graphene conductors
Source: Sci Rep. 2017 Aug 22;7:9052. doi: 10.1038/s41598-017-09465-x (PMC5567339; doi:10.1038/s41598-017-09465-x)
Supplement: Supplementary file 1 — Supplementary material [file 41598_2017_9465_MOESM1_ESM.doc]

**Increasing the doping efficiency by surface energy control for ultra-transparent graphene conductors**

**Kai Wen Cheng^1^, Ya-Ping Hsieh^2^,** **Chu-Chi Ting^2^, Yen-Hsun Su^1^ and Mario Hofmann^3*^**

^1^ Department of Material Science and Engineering, National Cheng Kung University, Tainan, 70101, Taiwan

^2^ Graduate Institute of Opto-Mechatronics, National Chung Cheng University, Chiayi, 62102, Taiwan

^3^ Department of Physics, National Taiwan University, Taipei, 106, Taiwan

*E-mail: [mario@phys.ntu.edu.tw](mailto:mario@phys.ntu.edu.tw) ;

**Supplementary Discussion**

Derivation of concentration dependent work function

The amount of transferrable charge from the AuCl_3_ depends on its work function difference to graphene ($\Delta W_{Au/G}$) and its geometric capacitance $C_{Au}$ according to

$$Q_{tr}=C_{Au}\Delta W_{Au/G}$$

The charge transfer process between AuCl_3_ and graphene equilibrates at a work function $\Delta W$

$$Q_{tr}=C_{Au}\Delta W+C_{G}\Delta W$$

, where $C_{G}$ is the capacitance of graphene.

We assume a simple proportionality between AuCl_3_ capacitance and the AuCl_3_ concentration ($c_{AuCl3}$)

$$C_{Au}=\alpha c_{AuCl_{3}}$$

We arrive at

$$C_{Au}V_{Au}=C_{Au}V+C_{2}V$$

$$\Delta W=\frac{\alpha c_{AuCl_{3}}\Delta W{}_{Au/G}}{\alpha c_{AuCl_{3}}+C_{G}}$$

The work function change of graphene can be calculated from its Hall carrier concentration n according to

,where v_F_ is the Fermi velocity.

The resulting fit is shown in Figure 1(d) with the extracted parameters

$$\Delta W_{Au/G}=0.7eV$$

$$\alpha=9.1mM^{-1}$$

$$C_{G}=7.6$$

Figure of Merit


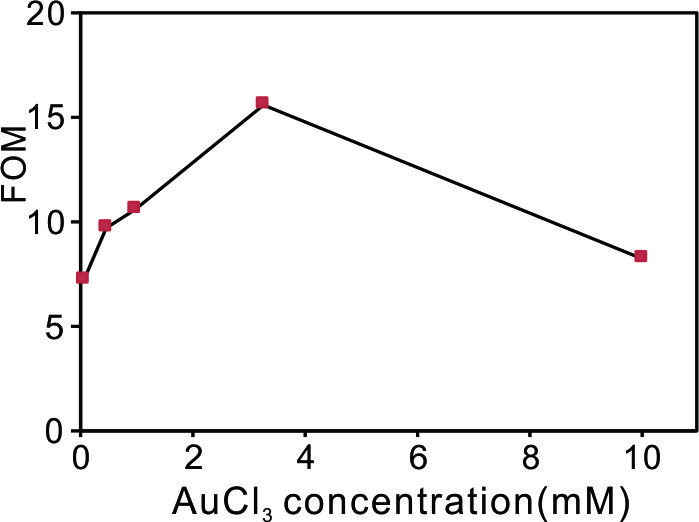


**Supplementary Figure S1.** Figure of Merit (FOM) vs AuCl_3_ concentration

Characterization of graphene


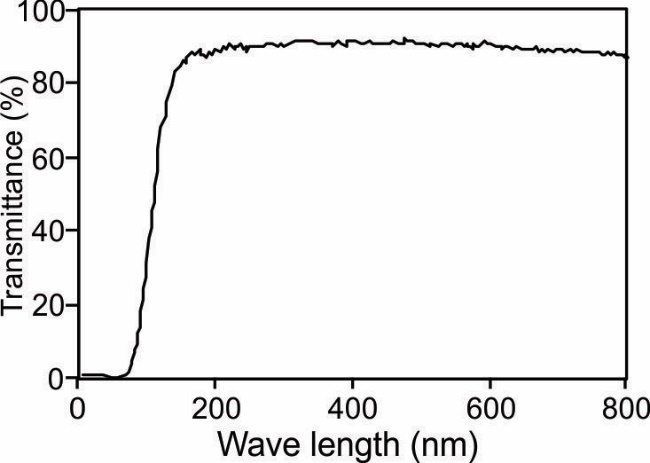

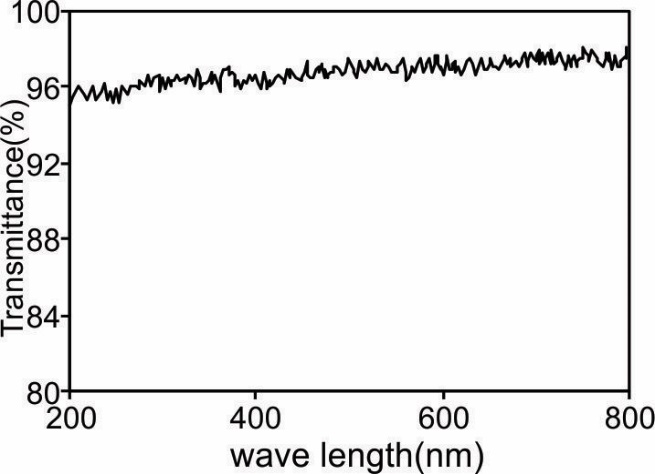


**Supplementary Figure S2.** Transmittance spectra of the graphene films after optimized doping show a featureless curve that indicates that the properties of graphene are retained

Atomic force microscopy of annealed PMMA


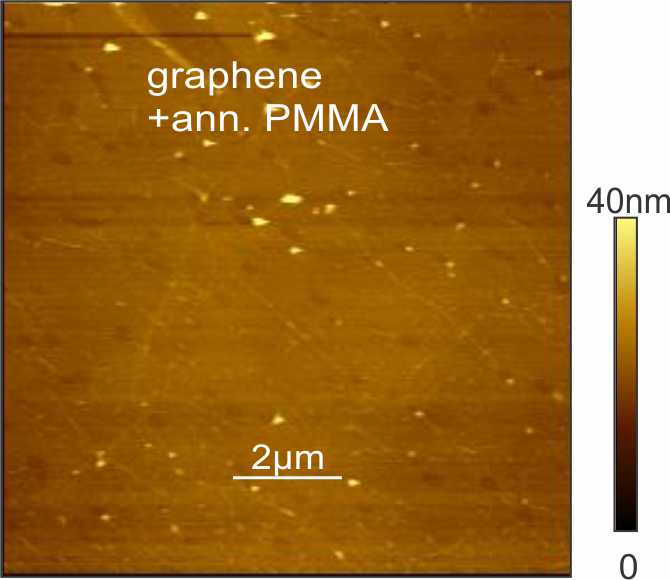


**Supplementary Figure S3.** AFM image of graphene with PMMA after annealing at 300°C shows little residue particles.

| **Process step** | **Average particle size** |
| --- | --- |
| Graphene | 22nm |
| Graphene+UV | 40nm |
| Graphene+annealed PMMA | 17nm |
| Graphene+annealed PMMA+UV | 70nm |

Overview of doping process


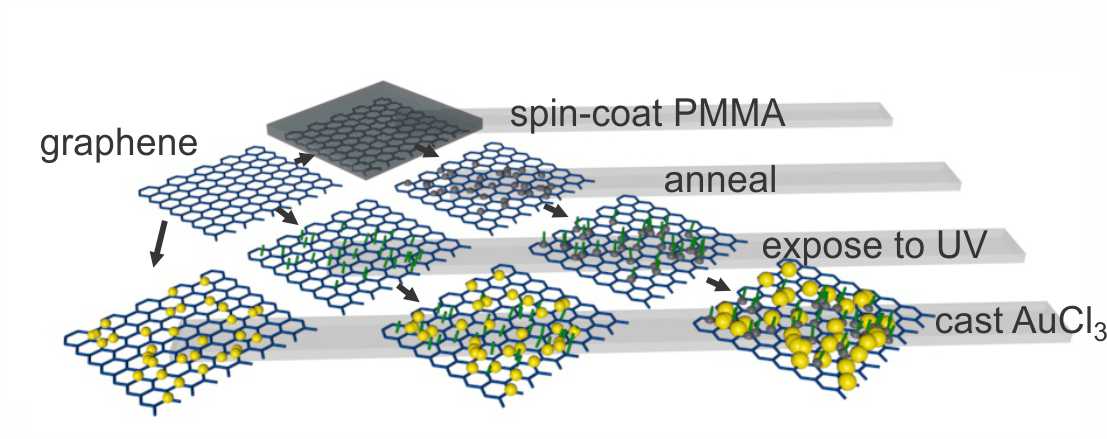


**Supplementary Figure S4.** Schematic of process steps carried out to arrive at 3 different AuCl3 doped sample types

In-situ characterization during UV exposure


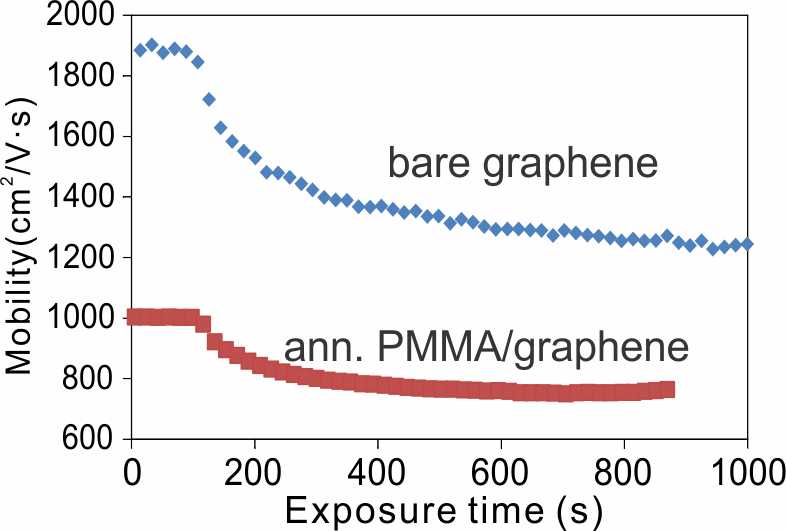


**Supplementary Figure S5.** The evolution of the Hall mobility with UV exposure is similar for bare graphene and graphene/PMMA stacks that were annealed at 300°C.

AFM characterization of final sample


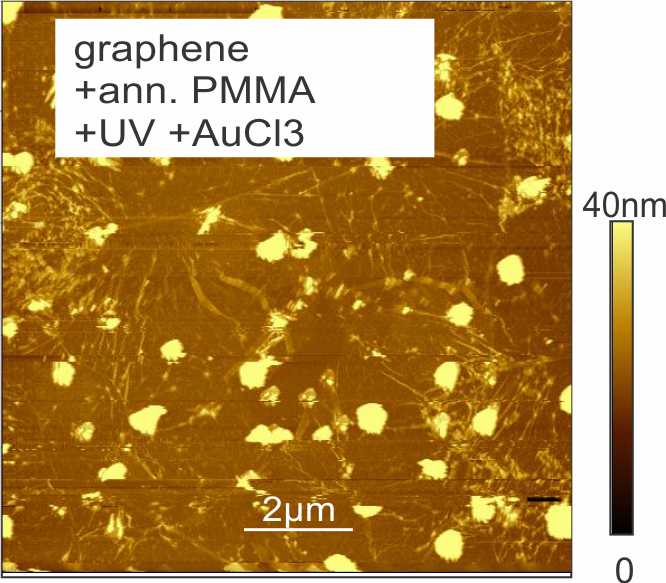


**Supplementary Figure S6.** AFM of a sample where graphene was coated with PMMA, annealed, exposed to UV, and AuCl_3_ doped shows the largest Au particle size.
